# Magnesium and Space Flight

**DOI:** 10.3390/nu7125528

**Published:** 2015-12-08

**Authors:** Scott M. Smith, Sara R. Zwart

**Affiliations:** 1 Biomedical Research and Environmental Sciences Division, NASA Johnson Space Center, Houston, TX 77058, USA; 2 Division of Space Life Sciences, Universities Space Research Association, Houston, TX 77058, USA; sara.zwart-1@nasa.gov

**Keywords:** microgravity, bone, tissue magnesium, bed rest

## Abstract

Magnesium is an essential nutrient for muscle, cardiovascular, and bone health on Earth, and during space flight. We sought to evaluate magnesium status in 43 astronauts (34 male, 9 female; 47 ± 5 years old, mean ± SD) before, during, and after 4–6-month space missions. We also studied individuals participating in a ground analog of space flight (head-down-tilt bed rest; *n* = 27 (17 male, 10 female), 35 ± 7 years old). We evaluated serum concentration and 24-h urinary excretion of magnesium, along with estimates of tissue magnesium status from sublingual cells. Serum magnesium increased late in flight, while urinary magnesium excretion was higher over the course of 180-day space missions. Urinary magnesium increased during flight but decreased significantly at landing. Neither serum nor urinary magnesium changed during bed rest. For flight and bed rest, significant correlations existed between the area under the curve of serum and urinary magnesium and the change in total body bone mineral content. Tissue magnesium concentration was unchanged after flight and bed rest. Increased excretion of magnesium is likely partially from bone and partially from diet, but importantly, it does not come at the expense of muscle tissue stores. While further study is needed to better understand the implications of these findings for longer space exploration missions, magnesium homeostasis and tissue status seem well maintained during 4–6-month space missions.

## 1. Introduction

Magnesium is a critical element, and its amount and concentration in the body have implications for many systems, including the cardiovascular and musculoskeletal systems, along with implications for the incidence of hypertension, stroke, migraine headache, diabetes, and other diseases [1,2]. Given the health risks of human space flight [3] along with a limited food system, adequate intake of magnesium is an important concern for ensuring astronaut health, especially on long-duration space missions.

One of the key issues with magnesium is that its status is not easily assessed, as is the case with many minerals. Serum and urinary magnesium are the most obvious variables to assess, but given that serum contains only 1% of the body pool of magnesium, changes (or lack of changes) in these measures do not necessarily reflect magnesium status [1,4,5]. Tissue magnesium concentrations provide a more direct, if not the best, assessment of magnesium status, and can be estimated through analysis of sublingual cells [5,6]. Due to limited availability and costs, implementation of this technique has been limited to date.

Magnesium is also important for bone health, and almost half of the body’s magnesium is maintained in bone [1]. Good-quality diets, rich in magnesium and potassium, have been associated with improved bone health [7,8]. Bone loss and negative calcium balance have traditionally been considered hallmarks of space travel [3,9,10,11,12], although recent evidence documents the ability of nutrition and exercise to mitigate bone mineral density losses after flight, albeit with remaining questions about the integrity and strength of the resulting remodeled bone [9,13,14]. Whether magnesium balance is similarly affected during space flight is unknown.

Magnesium assessments before and after space flight have been reviewed previously [10]. These assessments document a consistent drop in urinary magnesium after Apollo [15] and 4–6-month International Space Station (ISS) missions [16], along with small declines in serum or plasma magnesium after Apollo and Skylab missions [17], and slight increases in serum magnesium after early ISS missions [10]. Beyond the normal limitations of serum and urinary magnesium determinations, postflight assessments need to be examined cautiously given the fluid shifts that occur during flight, and fluid loading and recovery after flight. Some in-flight analyses from space missions of short duration (≤30 days) and up to 3 months, including some from crewmembers on Space Shuttle missions [10,18], have shown small declines in serum magnesium compared to preflight values. The decrease was likely not statistically significant (statistics were not performed due to the small *n* (2–6)), and the magnitude of change was also small (2.3%–8.3% below preflight values, on average) [10,18]. On Skylab missions, urinary magnesium increased during the first 2 months of flight, did not change during the third month, and decreased after flight [17]. 

Magnesium homeostasis is maintained through the balance of intake/absorption and renal excretion, with the latter playing a key regulatory role [4,5]. The menu for ISS crews contains 424 ± 40 mg magnesium/day (average ± SD) [10]. Dietary magnesium intakes on Shuttle missions (*n* = 32) were lower (294 ± 74) than on the ISS menu, which is not surprising given that overall energy intake was 74.2 ± 16 percent of estimated requirements for Shuttle astronauts [10]. While early ISS crews also often did not meet energy intake requirements, many crewmembers of late have maintained their energy intake and body mass during 4–6-month ISS missions [13,19].

Bed rest is the most commonly used ground-based analog to simulate the space flight effects on bone and muscle loss. One bed rest study showed slightly negative magnesium balance in untreated bed rest, with application of exercise mitigating this trend [20]. In other bed rest studies, urinary magnesium was unchanged during bed rest [21,22], but was significantly lower after bed rest [22], while estimates of tissue magnesium were unchanged [23].

We present here tissue magnesium concentrations before and after long-duration space missions, as well as urinary and serum magnesium from missions up to 6 months long. For comparison, magnesium status is also reported from bed rest studies up to 90 days. 

## 2. Experimental Section

### 2.1. Studies and Participants

Space flight data are reported from several studies and platforms. While some of the supporting data reported herein have been previously published, these are noted in the text throughout, and none of the prior publications have focused on magnesium. Protocols were approved by the National Aeronautics and Space Administration (NASA) Johnson Space Center Institutional Review Board (IRB), and informed consent was obtained from all subjects. Data from several bed rest studies are reported here, and again, many of these data have been published elsewhere [22,23,24,25], but none of the publications have focused on magnesium. The bed rest studies were approved by the NASA Johnson Space Center IRB, along with the University of Texas Medical Branch at Galveston’s IRB.

#### 2.1.1. Biological Sample Collections

Procedures for collection of biological samples from the space flight [13,26,27,28,29] and bed rest [22,28] studies reported here have been previously described in detail, and are reviewed here briefly.

#### 2.1.2. Space Flight

Data from 43 crewmembers (34 male, 9 female) who flew long-duration space flights (4–6-month missions) were used for these analyses. Two crewmembers each flew on two separate ISS missions, and on each mission each of these crewmembers was treated as an individual subject for the purposes of data analyses in this study. The mean age was 47 ± 5 years. Blood and two consecutive 24-h urine samples were collected about 180 and 45 days before launch, and again on landing day (return, or R+0 day) and 30 days after landing, as previously described [13,27,28,29]. Urine samples were available from 43 crewmembers and serum from 39. Pre- and postflight tissue magnesium samples were available from 33 of those subjects. When urinary and serum magnesium data were further analyzed by type of exercise (iRED or ARED) or exercise plus bisphosphonate, one less subject was used in those analyses due to the fact that one subject took bisphosphonates for only a part of the mission. Briefly, crewmembers provided five blood and 24-h urine collections during space flight at about flight day 15 (designated FD15), FD30, FD60, FD120, and FD180. Except for samples collected on R+0, all blood samples were collected at least 8 h after food intake or exercise. Because flight durations varied, not all crews had five in-flight sessions. Blood samples were collected using standard phlebotomy techniques, as described previously [14,26,27,28,30]. After centrifugation, tubes were frozen at −96 °C until they were returned to Earth (within 6 to 12 months) aboard the Space Shuttle. An additional blood sample was collected about 10 days before launch (L-10), and those tubes were frozen after centrifugation. The other preflight blood samples were aliquoted into smaller amounts before they were frozen, to avoid repeated freezing and thawing when the samples were analyzed. The in-flight samples were transported from the landing site to Houston, where they were stored in a −80 °C freezer until they were analyzed.

Space Shuttle 24-h urinary magnesium data were from 8 crewmembers on two missions (STS-121 and STS-115) of about 13 and 12 days’ duration. The 24-h urine collections were conducted 15–22 days before flight and immediately after landing. De-identified serum magnesium data from 504 crewmembers were obtained from NASA’s Life Sciences Data Archive.

Of the 33 ISS crewmembers evaluated for tissue magnesium status, 8 had access to the interim resistive exercise device (iRED) and 25 had access to the advanced resistive exercise device (ARED), which provided the capability for greater loads as previously described [13]. Of those 25 crewmembers, 6 took a bisphosphonate during flight as described [31]. 

Before and after flight, urine was collected into single-void urine containers (Cole-Parmer, Niles, IL, USA). Samples were stored with ice packs or refrigerated until they were processed, within 24 h of collection. In-flight urine voids were collected into urine collection devices containing 1 mL of a LiCl solution as a volume marker. These samples were processed as previously described [26,27,28,32,33].

### 2.2. Bed Rest

Twenty-seven bed rest subjects (17 male, 10 female) participated in 6-degree head-down-tilt bed rest studies for 60 to 90 days as described previously [22,34,35]. The mean age of the bed rest subjects was 35 ± 7 years old. Seventeen of these subjects were evaluated for tissue magnesium status. 

### 2.3. Biochemical Analyses

Blood and urine samples were analyzed for magnesium by ion-selective assay on the Olympus AU480 (Beckman Coulter, Brea, CA, USA) [14].

Tissue magnesium concentrations were measured in epithelial cells gently scraped from subjects’ sublingual mucosa [36], as we have previously described [23] for data from 12 of the 33 subjects reported herein. The sublingual cells were fixed on a carbon slide with cytology fixative and later examined with a scanning electron microscope to identify individual epithelial cells suitable for radiographic analysis (EXA; Intracellular Diagnostics, Inc., Medford, OR, USA). This method has been shown to be reliable, and one of the most accurate estimates of tissue magnesium status [5,36,37,38,39]. Reported tissue magnesium concentrations are the mean of 5 to 10 cells per subject, with normal population values being 37.9 ± 4.0 mEq/L [40].

Fractional magnesium excretion (FEMg) was estimated using the formula FEMg = [(urine Mg × serum creatinine)/(serum Mg × urine creatinine × 0.7)] × 100, where creatinine and magnesium are expressed as mg/dL [4]. As described by Topf and Murray [41], in the presence of hypomagnesemia, FEMg > 2% indicates inappropriate renal Mg loss and < 2% suggests that the hypomagnesemia is due to decreased intake or changed absorption. 

Bone mineral content was measured by dual-energy x-ray absorptiometry about 6 months before flight and within 1 month after flight as previously described [13,14], and within 1 week before and after bed rest [34].

### 2.4. Statistical Methods

All statistical analyses were performed using SigmaStat software. A one-way repeated-measures ANOVA with a Bonferroni post hoc test was used to assess change in serum and urinary magnesium over time. Student’s *t*-test was used to analyze Shuttle urine and serum data when only pre- and postflight data were available. All data in graphs are reported as mean ± standard deviation.

The area under the curve (AUC) for response to space flight (or bed rest) was determined for serum and urinary magnesium. The first data point used for the AUC determination was the average preflight value and the last was the data point for flight day 180. Pearson correlation coefficients were determined for serum and urinary AUC and the change in total body bone mineral content after flight. 

## 3. Results

### 3.1. Serum and Urinary Magnesium

**Figure 1:**
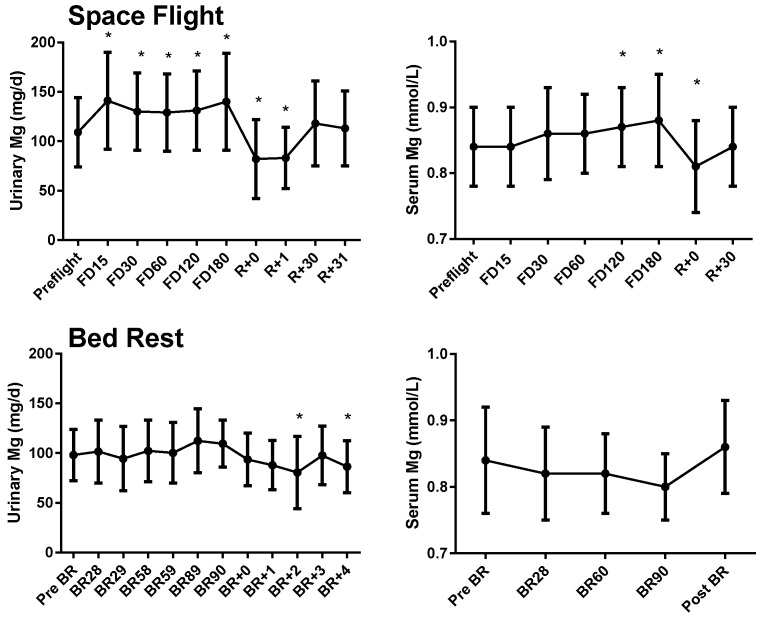
Urinary (*n* = 43) and serum (*n* = 39) magnesium before, during (flight days 15, 30, 60, 120, and 180), and after space flight (0, 1, 30, and 31 days after landing) (upper panels), and before, during, and after bed rest (lower panels, *n* = 27 urine and serum). Circles and error bars represent mean and SD respectively. * Significantly different from preflight mean (*p* < 0.05). For bed rest serum magnesium data, there was a significant effect of time (*p* < 0.05), but the post hoc Bonferroni test did not identify any individual group differences compared to baseline.

On 4–6-month ISS missions, 24-h urinary magnesium concentrations were significantly higher than preflight concentrations by flight day 15, and the elevation continued through the end of the mission (Figure 1). During the first 48 h after landing, urinary magnesium was lower than before flight, but it had returned to the baseline concentration when measured again about 1 month after landing (*p* < 0.05). When data were evaluated from the subset of crewmembers with pre/post tissue magnesium determinations (*n* = 33), the same pattern of increased urinary magnesium during flight and a decrease at landing was observed (data not shown). When serum magnesium was evaluated for the same subset of subjects, there was a trend for higher serum magnesium during flight, but significance was only observed at the last in-flight time point (FD180) and there was a decrease at landing. When data from all available crewmembers were included (*n* = 39), serum magnesium was increased by flight day 120 and remained increased until the end of the mission (Figure 1, *p* < 0.05). Similar to the urine data, serum magnesium was lower than preflight values on landing day but returned to baseline after 1 month. When serum magnesium was separated by groups (iRED, ARED, ARED+Bis), it was significantly higher at FD15 in crewmembers who took bisphosphonates compared to baseline and higher on FD180 among ARED subjects and lower on landing day and R+30 in ARED subjects compared to baseline (Supplemental Material: Figure 1). While these differences were statistically significant, all data were within normal ranges, and the magnitude of change would not be considered clinically significant.

**Figure 2:**
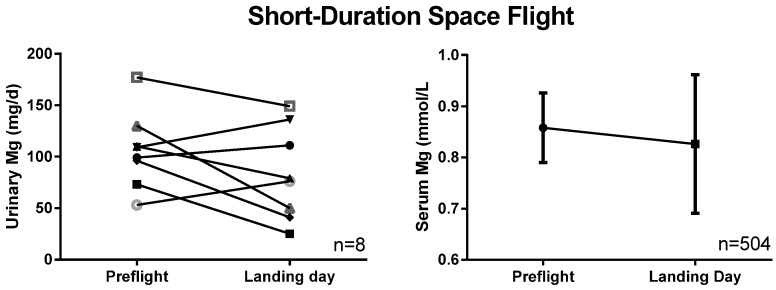
Urinary and serum magnesium after 2- to 18-day Space Shuttle flights. The urine data are individual data from a small study evaluating bone and mineral metabolism after flight, whereas the serum data are means ± SD from routine pre- and postflight medical testing, and thus have a much larger *n*.

In crewmembers on two short-duration Space Shuttle missions (*n* = 8), postflight 24-h urinary magnesium was no different from preflight (Figure 2). For a much larger (*n* = 504) cohort of Space Shuttle astronauts, no significant change occurred in serum magnesium after flight (Figure 2).

During bed rest, urinary magnesium was not different from the pre-bed rest baseline; however, 24-h urinary magnesium decreased on the second and fourth day after reambulation (Figure 1, *p* < 0.05, *n* = 27). Serum magnesium was unchanged during and after bed rest. There was a significant effect of time (*p* < 0.05), but a post hoc Bonferroni *t* test did not identify any individual differences from baseline (Figure 1). When a subset of subjects (*n* = 17) from whom tissue magnesium data were obtained were analyzed, urinary magnesium was decreased from baseline 2 days after reambulation and serum magnesium was lower than baseline after 3 months of bed rest (*p* < 0.05, data not shown).

**Figure 3:**
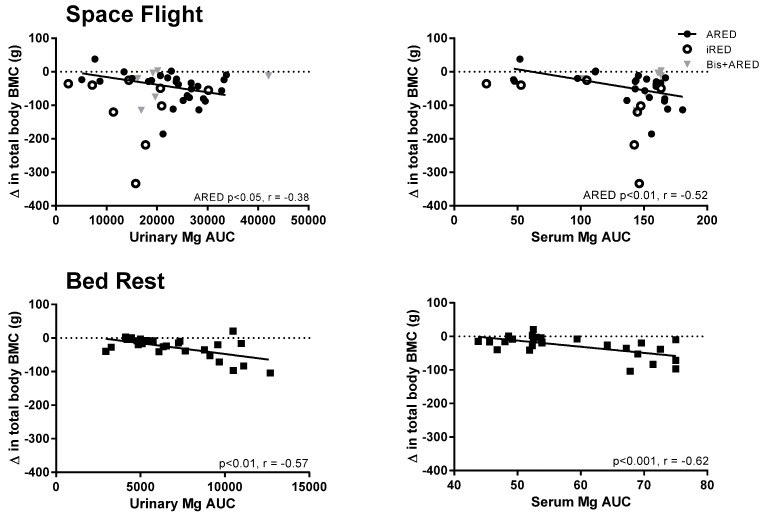
The response of urinary (*n* = 42) and serum (*n* = 38) magnesium during flight, expressed as the area under the curve (AUC), is related to the change in total body bone mineral content (BMC) after flight on the International Space Station (upper panels), and the AUC of urinary and serum Mg after 28 to 90 days of bed rest (*n* = 27 urine and serum) is related to the change in total body BMC after 60 to 90 days of bed rest (lower panels). Each circle represents an individual crewmember. In the upper panels, filled circles represent crewmembers who had access to the Advanced Resistive Exercise Device (ARED) and open circles represent crewmembers who had access to the interim resistive exercise device (iRED). Crewmembers treated with the bisphosphonate alendronate are shown with gray triangles. Pearson correlation coefficients are presented for significant correlations.

In a linear regression analysis, urinary and serum magnesium AUC during flight were both significantly related to the change in total bone mineral content after flight (Figure 3). The linear regression for urinary magnesium AUC was significant for crewmembers with access to the ARED but not those with access to the iRED or bisphosphonates (*p* < 0.05 and *p* < 0.01, respectively, for urinary and serum magnesium). Similarly, urinary and serum magnesium AUC during bed rest were both significantly related to the change in bone mineral content after bed rest (*p* < 0.01 and *p* < 0.001, respectively). 

Fractional excretion of magnesium (FEMg) was not different during flight, but it decreased significantly (*p* < 0.05) immediately after flight (Supplemental Figure 2). FEMg is typically used to evaluate magnesium loss in hypomagnesemia. While no subjects had serum magnesium meeting the definition of hypomagnesemia (*i.e*., < 0.7 mg/dL), even when the lowest tertile of serum Mg was compared to other tertiles, there was no difference in FEMg between these groups.

### 3.2. Tissue Magnesium

**Figure 4:**
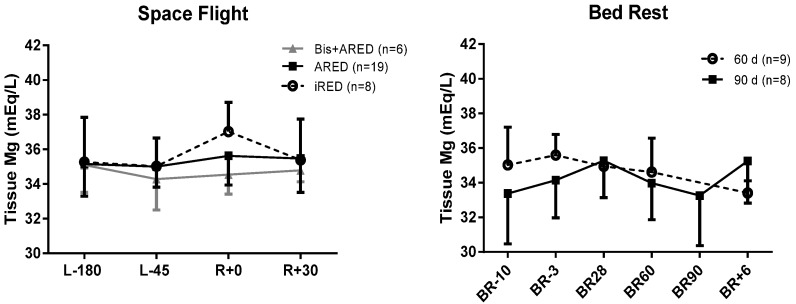
Sublingual tissue magnesium 180 and 45 days before flight (L-180 and L-45), on landing day (R+0), and 30 days after landing (R+30) (left panel). Crewmembers had available for exercise a cycle ergometer, a treadmill, and a resistive exercise device—either the interim resistive exercise device (iRED) or the Advanced Resistive Exercise Device (ARED) [13,14]. Six subjects participated in a bisphosphonate study in which they took an oral dose of 70 mg alendronate weekly during flight [31]. There were no significant differences among groups or after flight. In the right panel, data are from 17 subjects participating in 60- or 90-day bed rest studies 10 or 3 days before bed rest (BR-10 and BR-3), on bed rest days 28, 60, and 90, and 6 days after reambulation (BR+6).

For ISS crewmembers, sublingual tissue magnesium concentration after flight was not different from the preflight (Figure 4), and there was no effect of exercise type or bisphosphonate use. 

Sublingual tissue magnesium concentration did not change during bed rest for subjects participating in either the 60-day or the 90-day bed rest study (Figure 4).

## 4. Discussion

This is the first comprehensive evaluation of magnesium and space flight. We document here changes in serum and urinary magnesium during and after space flight and similarly in ground-based analogs. The key finding is that tissue magnesium is not affected by space flight, and that while some magnesium is likely lost from bone, there does not appear a need for overarching concern about magnesium deficiency during 4–6-month space missions.

Astronauts on long-duration ISS missions had increased serum and urinary magnesium during flight, which quickly reversed after landing. This postflight drop has been noted in previous long-duration space flight studies on Skylab and early ISS missions [16,42]. While it is well documented that serum and urinary magnesium are imperfect measures of magnesium status [1,4,41,43], these findings highlight the need for in-flight research, and the need for caution in interpreting postflight data with the intent of extrapolating to the in-flight condition. 

Potential sources for the increased serum magnesium concentration and urinary magnesium excretion during flight are bone, muscle and soft tissue, and diet. Evidence for bone resorption as a source of increased magnesium loss are the data showing that the AUCs of the serum and urinary magnesium responses to space flight were correlated with the change in bone mineral content after flight (Figure 3). While this relationship was statistically significant only for astronauts who had access to heavier resistance exercise loading with the ARED, the smaller number of data points available from iRED crewmembers followed a similar pattern, and a few outliers likely marred the statistical evaluation. Crewmembers treated with bisphosphonates, pharmaceuticals that block osteoclast bone-resorbing activity, had urinary and serum magnesium results very similar on average to the results for other crewmembers (ARED and iRED), albeit irrespective of bone loss. These data suggest that bone is the likely source of the magnesium response (*i.e.*, AUC) for amounts above that of the ARED+Bis crewmembers. Crewmembers in the ARED+Bis group had increased serum and urinary magnesium, along with the inability to resorb bone. From these data, we maintain that the basal magnesium response (AUC) is sourced from dietary magnesium. Given that ARED and ARED+Bis crewmembers consumed more energy than iRED crews [13], they likely had higher dietary magnesium intake, which would also help account for these data.

Bed rest did not yield a significant increase in urinary or serum magnesium, which would have been expected as some bone loss occurred in those subjects, albeit less than in astronauts [9,44,45]. Nonetheless, the AUC of the serum and urinary magnesium response to bed rest was linearly related to the change in whole-body BMC after 60–90 days of bed rest, and was less than the response observed during space flight (Figure 3). This suggests that a relationship existed between bone and magnesium loss during bed rest, but not at the magnitude where increased serum or urinary concentrations were evident. This likely resulted in part from the dietary magnesium intake during bed rest being generally below the RDA, and moreover being lower during bed rest than before bed rest (secondary to a reduction in overall energy consumption) [46]. 

Our findings highlight the need for caution in interpreting changes in magnesium status from blood and urine samples collected only before and after flight. Well-documented fluid shifts, and the physiological and/or intravenous replacement of circulatory volume in the hours and days after flight, clearly affected postflight serum and urinary magnesium concentrations, but not estimates of tissue magnesium. Magnesium could be a concern with respect to astronaut health, especially during high-stress mission phases such as reentry, extravehicular activity (EVA), and landing. The fact that tissue magnesium was maintained soon after landing, in the face of significant flux in circulating and excreted magnesium, provides reassurance that homeostasis protects critical muscle tissue stores.

In addition to high-stress mission phases, exercise effects on magnesium status are also a potential concern for astronauts. The large amount of exercise performed by space crewmembers [47] generally benefits astronaut health. Besides using the resistive exercise devices mentioned before, crewmembers perform aerobic exercise regularly on a treadmill and a cycle ergometer. The effects of exercise on magnesium balance (and other minerals for that matter) is not clear, and reports in the literature are inconsistent and contradictory [48]. Sweating in microgravity brings its own oddities, with no gravity assistance for dripping, and no convection for air cooling [49]. Concerns about thermoregulation during space flight have been raised [50,51], given the likelihood that the lack of water removal from the body may suppress cooling and increase heat load. Nonetheless, given the data presented herein, and the lack of change in tissue magnesium status after flight, there is no reason to conclude that increased loss of magnesium in sweat during flight is a concern.

Magnesium is of specific concern with respect to cardiovascular health [1,5,52], and there have been many studies showing the benefits of adequate (and/or supplemental) magnesium for the cardiovascular system, and conversely concerns about the possibility that magnesium deficiency could lead to increased incidence or severity of cardiovascular disease. The situation is confounded by the fact that (common) treatment of hypertensive patients with diuretics will result in increased magnesium loss. While evidence exists that blood pressure is reduced with magnesium supplementation [53], it is worth noting that the foods that are rich in magnesium (e.g., vegetables, whole grains) are also recommended for health benefits, in part because they are rich in other nutrients and phytochemicals as well. Kupetsky-Rincon and Uitto have noted that additional work is required to better understand magnesium intake in relation to health and disease, along with further evaluation of safety and efficacy of magnesium supplementation [52]. Further evaluation of the relationships between magnesium status and space cardiovascular data, and especially data obtained before and after exercise and EVA conditions, is nonetheless warranted. To date (and as those reported here), blood samples collected during space flight are typically collected at least 8 h after exercise, and days to weeks from EVA activity. Existing data should be carefully evaluated in light of these conditions.

Magnesium has been implicated as playing a role in other medical issues as well, including inflammation and insulin/glucose homeostasis. We and others have documented cytokine changes during space flight [54,55], but we have not found any relationships between magnesium status (serum, urinary, or tissue magnesium) and cytokine concentrations (data not presented). We did not determine catecholamines in the studies reported here, but recent studies in some of the same astronauts have shown no difference in plasma epinephrine or norepinephrine during or after flight compared to seated preflight values, despite the fluid shift to the upper body during flight that would be expected to suppress sympathetic nervous activity [56]. On short-duration Space Shuttle missions, urinary catecholamine excretion was lower during flight [57], and sympathetic nervous system activity was generally maintained during flight, with no significant differences [58]. Thus, no major alterations have been found in sympathetic nervous system control or activity, but clearly, this conclusion is derived from a limited set of findings, without regard to changes during exercise.

While results of experiments have suggested that glucose metabolism and insulin insensitivity are altered in space flight [59,60] and in bed rest [61,62,63], these suggestions came from missions or studies in which little or no exercise was conducted. ISS crewmembers typically exercise 6 days per week, using a combination of aerobic and resistive exercise devices [47], and we and others have hypothesized that this regimen will likely normalize glucose metabolism. Given our data documenting no changes in tissue magnesium concentrations after landing, there is little reason to speculate that magnesium deficiency may be causing altered carbohydrate metabolism. Nonetheless, additional studies of carbohydrate metabolism during long-duration space flight with nominal exercise profiles would provide data to resolve this question.

On Earth, and there is little reason to think the situation different in space, a clear relationship exists between energy intake and magnesium intake. Thus, dietary intake of magnesium is a matter of concern for astronauts, but largely within the overarching concern of adequate dietary energy intake [10,16]. In athletes, it has been documented that magnesium intake is maintained, except in athletes given to energy restriction (e.g., dancers, wrestlers, gymnasts) [64]. Although a detailed evaluation of magnesium intake during space flight has not been conducted, energy intake is tracked and crews are actively encouraged to maintain body mass during flight. Recent ARED crews have shown that it is possible to consume adequate caloric intake and maintain body mass [13]; thus we expect that magnesium intake is adequate given we know the menu contains an adequate amount [10]. Again, additional study of dietary intake, as currently planned for future ISS missions, would resolve this question with data.

Magnesium homeostasis is a balance between absorption from the diet and renal excretion of magnesium, with renal excretion being the key regulatory component, varying to accommodate influx from the intestines and other endogenous sources [43]. The only magnesium balance study we are aware of that was conducted during space flight was on the 14-day Gemini-VII mission [65]. Magnesium balance in these two crewmembers was negative, and was about 100 mg/day lower during flight than before flight. Dietary magnesium intake was also lower during flight (198 ± 40 mg/day for each crewmember, mean ± SD) compared to preflight intake (388 ± 11 mg/day and 366 ± 18 mg/day for the two crewmembers). These in-flight intakes are much lower than current dietary recommendations of 420 mg/day for men 31–50 years old [43]. To our knowledge, only one study has evaluated magnesium balance during bed rest [20]. That report included only the net balance data, not the individual components (e.g., dietary intake, urinary and fecal excretion). In the first 120 days of bed rest, the authors found negative magnesium balance in both the control (−58 ± 21 mg/day, mean ± SEM, *n* = 5) and exercise + bisphosphonate (−65 ± 13 mg/day, *n* = 4) treatment groups. In the subsequent 120-day period, the control subjects exercised, and both groups’ magnesium balance increased toward pre-bed rest levels and was close to or above zero by the third 120-day period (Mg balance for the exercise-alone group was −5.4 ± 8 mg/day, and for the exercise + bisphosphonate group it was positive +23 ± 27 mg/day). These data suggest that the initial negative magnesium balance had little to do with bone loss, and that this effect recovers with time. The authors attributed these results to dietary intake, and the fact that while participants were exercising, their intakes of magnesium were greater than during the sedentary control period. While available bed rest data are described herein, a more comprehensive evaluation of magnesium balance and absorption in a bed rest model, with and without exercise countermeasures, would provide valuable information.

We report here tissue magnesium during sedentary bed rest, but have not evaluated this variable in bed rest studies evaluating exercise countermeasures. Although sublingual cell analysis is a valuable technique, it is limited in that only one commercial lab provides these determinations and in-flight determinations cannot easily be made because of required real-time sample processing. For another approach, adapting magnesium stable isotope analysis techniques, as we have done with calcium [66,67,68], could provide valuable insight into magnesium homeostasis, and could ultimately lead to a noninvasive clinical tool to better evaluate magnesium homeostasis. 

Magnesium deficiency and hypomagnesemia are most often accompanied by hypocalcemia and hypokalemia, typically secondary to diuretic use [69]. We have never observed simultaneous alterations in magnesium, calcium, and potassium in our data from ISS crewmembers. Furthermore, we have never observed hypomagnesemia (serum magnesium < 0.7 mmol/L) in any ISS astronauts to date. Nonetheless, the deficiencies typically seen on Earth with diuretic use serve as a cautionary tale for flight surgeons considering the use of diuretics in their astronaut patients.

Limitations of the studies reported here include the ever-present inherent difficulties in space research. While the subject numbers here are respectable relative to those in much of the space science literature, a larger sample set, including larger subgroups (e.g., sex and race) would aid extrapolations to the general population. In-flight collection of sublingual scrapes would enhance the dataset, although this was precluded by sample collection and preservation techniques.

In summary, magnesium is a critical nutrient, with many important functions. Although on Earth magnesium homeostasis can be altered by disease states and medication use, the data presented here document that despite consistent and significant alterations in urinary magnesium after space flight, tissue stores of the mineral are maintained, and in-flight data reveal increased serum and urinary magnesium. Thus, we maintain there is no general cause for concern about magnesium deficiency in astronauts. Additional studies are warranted, as discussed here, to better understand the role of magnesium in astronaut health and to better define the nutritional requirement for magnesium in astronauts. Findings from such studies, as with most NASA-related research, may also help inform those interested in similar questions about magnesium for those remaining on Earth.

## Supplementary materials

Supplementary materials can be accessed at: http://www.mdpi.com/2072-6643/7/12/5528/s1.
