# Supplementary material for: Magnesium and Space Flight
Source: Nutrients. 2015 Dec 8;7(12):10209–22. doi: 10.3390/nu7125528 (PMC4690080; doi:10.3390/nu7125528)
Supplement: Supplementary file 1 [file nutrients-07-05528-s001.docx]

Supplementary Materials: Magnesium and Space Flight

**Scott M. Smith ^1,^* and Sara R. Zwart ^2^**

**Figure S1.** Serum and urine magnesium before, during (flight days 15, 30, 60, 120, and 180), and after (return plus 0, 1, 30, or 31 days) space flight in crewmembers with access to the interim resistive exercise device (iRED), advanced resistive exercise device (ARED), and those who took the bisphosphonate alendronate once per week during flight (Bis+ARED). For serum, there was a significant interaction effect (*p* < 0.05) in that the Bis+ARED group was higher at FD15 than baseline and the ARED group was higher than baseline at FD180 and lower than baseline at R+0 and R+30. For urine, all groups were higher at FD15 and lower at R+0 and R+1 (*p* < 0.001).

**Figure S2.** Fractional excretion of magnesium (FEMg) before, during (flight days 15, 30, 60, 120, 180), and after (return plus 0, 1, 30, or 31 days) space flight. FEMg was estimated using the formula
FEMg = [(urine Mg × serum creatinine)/(serum Mg × urine creatinine × 0.7)] × 100, where creatinine and magnesium are expressed as mg/dl [4]. FEMg was significantly lower on landing day and in the first 24 h after landing.
